# Use of an evidence-based health portal to improve teaching and learning in primary care: a mixed methods evaluation

**DOI:** 10.1186/s12909-021-02686-9

**Published:** 2021-05-24

**Authors:** José Roberto Bittencourt Costa, Luiz Anastacio Alves, Anael Viana Pinto Alberto, Cristina Alves Magalhães de Souza

**Affiliations:** 1 Undergraduate Course in Medicine, University Center Serra dos Órgãos (UNIFESO), Cellular Communication Laboratory Postgraduate Program in Teaching in Biosciences and Health, Fundação Oswaldo Cruz (FIOCRUZ), Rio de Janeiro, Brazil; 2 Cellular Communication Laboratory, Oswaldo Cruz Institute (IOC), Oswaldo Cruz Foundation (FIOCRUZ), Rua Leopoldo Bulhões 1480, Pavilhão 108, sala 28B, Rio de Janeiro, Brazil

**Keywords:** Medical Schools, Family Health, Evidence-Based Medicine, Portals for Access to Scientific Journals

## Abstract

**Background:**

Worldwide, primary care is for most people the gateway into many health systems. Offering solutions to the demands of the communities served requires the constant preparation of professionals, especially doctors and medical undergraduate students. We analyze and propose ways to improve the teaching and learning processes facilitated by the Basic Family Health Units (BFHUs) based on the use of electronic portals with evidence-based medicine criteria.

**Method:**

First phase: The authors conducted a qualitative-quantitative study on students and instructors of primary care (PC) medicine by administering a survey of open- and closed-ended questions at medical schools. The closed-ended questions were studied with descriptive statistics, and open-ended questions were analyzed via the creation of categories. Perceptions of major teaching and learning problems were then identified.

Second phase: Meetings were held with students and their instructors for 6 months and involved the use of electronic portals and the application of new questionnaires using a Likert scale for pre- and postevaluation.

**Results:**

In the first phase, 40% of the students considered local instructor training levels a problem. A similar result was found regarding teachers’ lectures, revealing a lack of adequate PC training and performance.

Building on our results, we focused on BFHUs to apply new strategies for teaching and learning, such as the use of the Evidence-Based Health (SBE) Portal, which includes several databases with clinical evidence criteria. In the second phase, the authors identified an improvement in the quality of learning among instructors and students. This outcome improved safety in daily clinical practice in PC, possibly with better results for its users.

**Conclusions:**

The use of electronic portals can facilitate BFHU teaching and learning and promote the health of users.

**Supplementary Information:**

The online version contains supplementary material available at 10.1186/s12909-021-02686-9.

## Background

In 2017, the World Federation for Medical Education (WFME) estimated that there were approximately 2900 medical schools around the globe (https://wfme.org/world-directory/), with Brazil having the second-largest number of schools, with 336, behind India (392) (https://www.escolasmedicas.com.br/escolas-medicas-brasil-e-internacionais.php). Despite the number of medical schools in Brazil, integrated health care, which takes biopsychosocial aspects into account and provides care geared to the reality of each family or individual, is far from being used in everyday medical practice. The use, for example, of expensive treatments outside the real context of users of the health system is still frequent [1, 2].

While medical schools may seem similar, they differ considerably in their curricula and in the types of training they provide.

Medical schools should consider the needs of health system users, understanding them in their integrality, that is, acknowledging the process of illness as inextricably connected to their lives. As an example, in the treatment of scabies, it is not sufficient to focus on medication to eliminate the causal agent, *Sarcoptes scabei*, with a scabicidal agent. It is also necessary to propose changes in personal hygiene and lifestyle, considering the ability of each patient to implement them. It is essential, therefore, to propose ways to improve teaching and learning processes that are contextualized to real locoregional demands, and this need was what effectively guided the study presented here [3, 4].

Therefore, the practice of medicine should adopt modes of knowledge production and academic training compatible with the real life circumstances of patients [4, 5].

In this context, the diversification of the teaching-learning scenario offered by primary care (PC) is presented as a timely reference for a medical practice driven by social responsibility and improvement of the vision of integrality. In the PC setting, the student can experience the real socioeconomic limits of the population and how users access health care [6].

Despite the perspectives previously mentioned, the experience of students in PC presents different tensions, as reported by Gil et al. (2008): (a) “little time to practice with students due to the demands of the community”; (b) students' feeling that they "hinder services" when they do not have the skills to contribute to care; and (c) the loss of the practice of home visits to the detriment of the diagnosis of health problems in the community [7, 8]. Costa (2009) found that the Basic Family Health Units (BFHUs) could support the teaching-learning process if there was better planning regarding the teaching and the activities offered in these units more qualified health professionals and teachers [9]. Ciuffo et al. (2008) and Trajman et al. (2009) identified gaps in teacher training, including regarding teaching methodology use, the availability of extended clinical courses and specializations in family health, and the lack of salary increases [10, 11]. How, then, can we improve PC teaching/learning practices?

Based on answers collected from a qualitative and quantitative survey of students from four Brazilian medical schools and their respective PC instructors, we propose the application and analysis of a new teaching-learning strategy, constituting the main aim of this study. In this context, we examine evidence-based medicine (EBM) because it plays a fundamental role in clinical decision-making in several medical specialties and because it uses statistical estimates of benefit-risk and harm for decision-making purposes [12, 13].

## Method

First, it was necessary to verify which aspects related to the teaching-learning process in the health units could be improved. Then, an electronic tool was offered that could facilitate the work of doctors and students as described below:

### Investigation

Qualitative study of PC students and instructors who work in community-based clinical preceptories and analysis of the main problems in teaching-learning under such conditions and of possible means of improvement (Additional file 1: Appendixes A, B and C).

### Implementation of a coherent research strategy

Trial implementation of a teaching-learning strategy according to the results identified in the first phase of research (Additional file 1: Appendixes D1 [pre] and D2 [post] and E1 [pre] and E2 [post]).

Two research phases were applied:

- **First phase (investigation)**

The study was carried out in a private medical school, the Centro Universitário Serra dos Órgãos (UNIFESO), in Teresópolis, Rio de Janeiro (RJ), and in three federal schools: Universidade Federal Fluminense (UFF), RJ, Universidade Federal do Tocantins (UFT) and the Universidade Federal of Viçosa (UFV). These four schools are designated medical schools A, B, C and D. All of them are located in urban areas. The criteria for selecting these schools were based on the accessibility and availability of teachers/instructors and their students. In addition, a curricular model focused on comprehensiveness and student insertion in PC/BFHUs or new practice scenarios was taken into consideration. The study population included students and instructors with 1 year of experience in PC/BFHU operation. We distributed questionnaires with 17 closed-ended questions to 237 students enrolled in 4 medical courses. The questions addressed the following topics: (a) motivations for choosing a medical career; (b) perceptions of the curriculum adopted at the studied school; (c) factors affecting participation in PC/BFHU activities, the main problem affecting teaching and learning, and suggestions for improvement; and (d) the influence of participation in PC/BFHUs in terms of their professional trajectory.

For the purposes of this research, only 2 questions were selected (Additional file 1: Appendix A: questions 10 and 11; Appendix B: questions 11 and 12): open-ended questions on the main problem affecting PC teaching-learning and on suggestions for improvement based on 10 options. These options were ranked with values of 1 (high priority) to 10 (low priority).

The 32 preceptors’ (08 from A, 13 from B and 14 from C) answers to the questionnaire with 4 open-ended questions were analyzed with a focus on a question exploring their views on BFHU teaching (see Additional file 1: Appendix C).

The questionnaires applied above were validated in pretests with 10 students and 05 teachers, where the level of understanding of the questions asked and the answers obtained were verified.

The samples presented here were selected by probabilistic sampling or randomly based on criteria of convenience, accessibility and similar responses (i.e., data saturation). In this way, 237 students and 32 teachers were recruited. Therefore, it was considered that the representativeness, homogeneity, and quality of the information obtained was sufficient to extract new information or ideas. Then, we applied descriptive statistical analysis to the answers to the closed questions and categorization of the answers to the open questions [14–19].

Simple random sampling involves assigning each element of a population a unique number and then selecting some of these elements at random. To ensure that a sample is selected randomly, random number tables can be used. Such tables include numbers presented in columns or consecutive pages. These guidelines were followed in our methodology to obtain the samples [7].

- **Second phase (implementation of a coherent research strategy)**

Then, questionnaires containing 13 assertions were given to the instructors and students to evaluate their use of electronic resources. In this way, it was possible to verify previous and acquired knowledge on EBM and on electronic portals on this theme in daily BFHU practice. These questionnaires were also validated in pretests to verify the understanding of 05 undergraduate students and 02 preceptors. The results were analyzed using a Likert scale pre- and postevaluation (Additional file 1: Appendixes D1 and D2 and E1 and E2) according to Norman (2010) and Phelps et al. (2015) [20, 21].

A score of 1 to 5 was established for each statement presented. The score increased based on the degree of previous or acquired knowledge relevant to the research and did not necessarily reflect agreement with the answers.

For the Likert scale scores, the maximum number of absolute points for each group/instructor was recorded: the product of the number of maximum points for each question, the number of questions included in each questionnaire applied (13 questions), and the number of participants completing each questionnaire. The relative number was based on the relation between the number of points obtained considering the possible points for each questionnaire and the respective number of participants [19, 20]. An open-ended question was also included at the end of the questionnaire together with the qualitative analysis questionnaire based on certain categories (Additional file 1: Appendix D1 and D2) [14, 15].

## Results

### First phase

#### Students

The students’ responses revealed that to improve teaching-learning in PC, *instructor training needed to be prioritized*, as this was mentioned in 91 responses (40%) of the 237 obtained. *A lack of instructor training* was deemed the most pressing issue, being cited in 83 responses (35%). These results were obtained in seven periods (classes) of different degrees from medical schools A, B, C and D. In two classes, the results achieved reached 51% for “instructor training” as a condition to be prioritized and 48% for “lack of instructor training” as the most serious problem. Other responses were randomly distributed through simple random sampling and were not nearly as prevalent as the above two responses [7]. The open-ended question was not answered with the option "other", indicating a different opinion.

#### Teachers

Our study of instructors' perceptions revealed several tensions in terms of PC, the lack of instructor specialization in family health and PC, inadequate planning of activities performed, and excessively large class sizes. The professionals pointed out *a lack of continuing education activities as a barrier to PC teaching-learning improvement*.

Table 1 shows the main issues raised by the instructors from 3 medical schools (A, B and C) in response to the question chosen for this study. Medical school D did not participate in the instructor survey.

**Table 1:** Themes identified from conversations with medical school A, B and C preceptor

Themes identified from conversations with medical school A, B and C preceptor	
How would you recommend improving teaching and learning in Primary Care or Basic Family Health Units (BHFUs)?	
Themes	**Responses from medical school A preceptors**
Community projects Learning through teaching planning Preceptor qualifications Permanent education and continued	"Maintaining contact with the community is very important for learning purposes" "The interns learn a lot by teaching the younger students" "A smaller number of students should be taught at a time" "A large number of students and population to be served limits the amount of time that can be dedicated to each individual" "Integrate the academy with city governance to improve practices" "Focus the academy more on real conditions" "I try to help identify themes and structure proposed schedules" "Specialists must advise us on the different Units" "We need continuing education activities"
Themes	**Responses from medical school B preceptors**
To learn is to do Preceptor qualifications Planning Preceptor professional development Emergency care reopening Early insertion Active learning methods	"The best way to learn is to practice doing tasks" "Knowledge from Unit specialists should be used to move beyond ethics to doctor-patient relationships and technical knowledge" "The teachers are specialists who engage in specialized HUAP^a^ activities and who do not want to work with the HBU^b^ because they do not want to leave the hospital environment. The university should have teachers with this profile work directly with the BHFU ..." "Supervision should be more active" "Preceptors should engage with professionals involved in services" "There must be a willingness to engage in TCS^c^" "The HBU^b^ must be structured to receive students and teachers and to understand what it means to incorporate a new discipline into the unit" "We must not forget the need to constantly read new articles" "Reopening emergency care at HUAP^a^ would be a great experience" "I think that insertion in later stages before graduation would be more productive" "Use active learning methods"
Themes	**Responses from medical school C preceptors**
Preceptor qualifications Planning Community projects	"There must be specialized academic training with specialized didactic material" "The HBU^b^ determines the number of people assisted and there is no time to discuss cases" "We must improve counter references for the discussion of diagnoses" "There is not enough discussion time" "There has been decline in local demand" "We should standardize subjects and apply activity-based learning" "We must provide more training in community interventions" "More time should be dedicated to carrying out community projects"

Source: the authors

^a^HUAP - Antônio Pedro University Hospital

^b^HBU - Health Basic Unit

^c^TCS - Supervised Field Work

The instructors’ responses frequently cite issues related to "planning" and "instructor qualifications.”

### Second phase

According to the results described in the first phase above, we found that to improve teaching and learning in the health units, it would be necessary to improve the training of the professionals who work there, in particular, the preceptor; thus, we elaborated our second question:

How can preceptors improve and be better trained?

We realized that the use of electronic smartphone-based tools in PC, both by the preceptor and the students, was frequent but that users accessed databases randomly without any consensus, generating a plurality of results and doubts, mainly regarding the behaviors in situations everyday clinics.

Therefore, to address the question above, we opted for the use of new electronic resources. Among these, the use of an electronic portal, which contextualizes criteria for evidence-based practice, resulted in the emergence of the second, and more productive, phase of this work described below.

We held workshops (Additional file 1: Digital Appendix F) on the following clinical issues relevant to BFHU students and instructors and daily BFHU practices. The themes were chosen freely and by common consent. Among these, we highlight the following: “Is physical activity beneficial for those who have already had an acute myocardial infarction (AMI)?”, “Who needs Aspirin®?”, “Beware of salt! Cigarettes? No way..."

We found that of the tools available, the Evidence-Based Health Portal (SBE) offered the best research resources for tutors and students via 12 databases and the Atheneu Library [22].

The portal offers access to 3 SBE databases, namely, Dynamed, Pro Quest and the British Medical Journal (BMJ), deemed by the students and instructors to be excellent sources of information on clinical studies based on (clinical) systematic reviews. The preceptor and the interns selected articles and systematic reviews using keywords related to the theme from a metasearch engine that analyzed the 12 databases offered by the portal simultaneously, including the 03 SBE databases.

During the first semester of 2013, approximately 20 workshops were carried out with 2 groups of 5 interns (G1 and G2) and with their respective BFHU instructors under the Secretary of Health of Teresópolis, RJ, located in a rural area.

A list of the workshops held and their respective themes and considerations is shown in Table 2.

**Table 2:** List of workshops held on selected themes and relevant comments

Selected themes and activities	Comments
Group G1 Period: 01/2013 to 04/2013	
Weekly workshops involving one preceptor and five interns. Pre- and post-test application using a Likert scale. Themes: Is prophylactic use of acetylsalicylic acid (Aspirin®) beneficial to heart disease patients? Is prophylactic use of Aspirin® beneficial to healthy individuals? Is physical activity beneficial for those who have had or not had an ^a^AMI? What is the relationship between smoking and cardiovascular disease? What is the relationship between diet and cardiovascular disease? What are the benefits of Omega 3 and 6 application? What are the benefits of prescribing polyvitamins and calcium for the treatment of osteoporosis and osteopenia? What are the benefits of applying a post-test?	Excellent reception. The proposal to discuss clinical issues has been accepted. ^d^EBM and Telehealth discussion. Themes chosen through group consensus among students and the preceptor. Discussion of practical issues such as TV show use. Rudimentary questions that include broader questions. Full use of the portal and its databases in addition to the ^b^BVS. Discussion of systematic reviews. Construction of a flowchart for accessing databases.
Group G2 Period: 04/13 to 07/13	
Weekly workshops involving one preceptor and five interns. Application of a pre-test using a Likert scale. Themes: What are the health benefits of zinc use? What benefits does zinc use offer in childhood, youth and adulthood? How can we use pumpkin seeds; phytotherapy and applications of folk, complementary or alternative medicine to address common complaints related to PC. Phytotherapy Vertigo: What is it? What is its etiology and diagnosis? How can one treat it? What is the efficacy of using cinnarizine (to treat dizziness) and Ginkgo biloba (a herbal medicine used to treat dizziness)? Application of a post-test using a Likert scale	Excellent reception. Discussion of clinical issues. ^d^EBM and Telehealth discussion. Themes chosen through group consensus among students and the preceptor. Full use of the portal and its databases in addition to the ^b^BVS. Use of systematic reviews and the ^c^SBE Portal. ^c^SBE is expanded to include 12 databases.

Source: the authors

^a^*AMI*: Acute Myocardial Infarction

^b^BVS: Virtual Health Library

^c^SBE: Evidence-Based Health

^d^EBM: Evidence-Based Medicine

After the workshops, we verified the following results on pre-and post-acquired portal information: 67% (40/60) of the instructors had used information in the preapplication research phase, and 92% (55/60) did so after applying the tool; 62% (220/325) and 76% (247/325) of the G1 and G2 groups, respectively, did so in the preapplication phase, and 92% (300/325) and 84% (272/325) of the G1 and G2 groups, respectively, did so in the postapplication phase. These results may signal a greater degree of knowledge acquisition because the higher the score obtained on the posttest, the greater the likelihood that knowledge has improved. Some of the questions used in this phase as well as the respective scores obtained, including the last statement - No. 13 - are shown in Table 3.

**Table 3:** Examples of some of the questions on the Likert scale of interns (10 students) and their preceptors with scores from 1 to 5 given for each statement and results obtained

Questions	PRE 10 interns (G1+G2) and 1 preceptor (Preceptor)			POST 10 interns (G1+G2) and 1 preceptor (Preceptor)		
	Prec.	G1	G2	Prec.	G1	G2
1) Primary Care teaching and learning should be improved	4	24	17	5	25	16
2) I have knowledge of search portals	4	12	17	5	22	21
3) I have knowledge of Telemedicine and Telehealth resources	4	8	8	4	24	19
7) I have knowledge of evidence-based medicine	1	12	18	4	23	24
8) I apply Evidence-Based Medicine in Basic Care	2	17	20	4	24	22
13) I discuss medical practices based on evidence-based medicine with my teachers or preceptors ^(**)^ ** For the preceptor = students	1	15	16	5	24	22
Total possible (absolute number including 13 questions)	**60**	**325**	**325**	**60**	**325**	**325**
Total presented (absolute number including 13 questions)	**40**	**220**	**247**	**55**	**300**	**272**
Total (relative number)	**67%**	**68%**	**76%**	**92%**	**92%**	**84%**

Source: the authors

Regarding the open-ended question on perceptions of the use of Internet tools, we identified the following focuses: "knowledge expansion," "applicability in practice," "the rate of information dissemination" and "updating". There were no distinctions between answers given by interns from groups G1 and G2 in either the pre- or postapplication phase. The instructors focused on one of the students’ answers: “the acquisition of knowledge."

We then verified studies on this subject listed in the Virtual Health Library (BVS). This database was selected due to its academic representation of teaching in the Brazilian health care field; representation within the BVS Network, which covers 30 countries in the Americas, the Caribbean, Africa and Europe; and quality, ensured through its certification by the Latin American and Caribbean Center on Health Sciences Information (BIREME)/Pan American Health Organization (OPAS)/World Health Organization (WHO).

For our study, we used 3 fundamental terms: "instruction," "training" and "basic care". The term "tutoring" was also used rather than "instruction" as a term already classified as a descriptor of health. The results presented here are based on 175 documents, including 171 articles and 2 theses. We chose to narrow our results by applying "tutoring" as a search term (leaving 90 documents) and then applying the "complete text" limitation, leaving 24 national and international documents (23 articles and 1 thesis). To better analyze these results, we summarize the content of certain studies in Table 4 [4, 11, 23–26].

**Table 4:** List of selected references with study titles and modalities and respective themes identified from the BVS research using the terms instruction and basic care

REFERENCES	
Title and type of study	-Objective of study with intervention proposal-
*Ensino e Aprendizagem em Serviços de Atenção Básica do* ^a^*SUS: desafios da formação médica com a perspectiva da integralidade. “Narrativas e Tessituras”* *PhD Thesis* [4]	Focus on teaching, learning and evaluation in basic ^a^SUS services with the analysis of obstacles to and potential for the transformation of training and assistance in terms of completeness with reference to the ^b^DCNs. The work recommends that new care projects for care and training be developed collectively.
*A preceptoria na rede básica da Secretaria Municipal de Saúde do Rio de Janeiro: opinião dos profissionais de Saúde* *Article* [11]	Evaluations of 351 PC health professionals of the ^c^SMS in Rio de Janeiro on teaching activity. It is shown that there has been little appreciation and encouragement of teaching based on work and teaching conditions or improvements of salaries, infrastructure and professional qualifications. The ^d^IES and the state are responsible for carrying out effective partnerships to mitigate this situation.
*Improving education in primary care: development of an online curriculum using the blended learning model.* *Article* [23]	Effectiveness of using a mixed learning curriculum at Case Western Reserve University’s School of Medicine in Cleveland, Ohio using modules available online.
*Estrategia de superación para perfeccionar la labor del tutor em los estudiantes de Medicina de la Filial de Ciencias Médicas de Morón.* *Article* [24]	The study finds that the training of tutors for general practitioner students remains insufficient at the School of Medical Sciences of Moron in Cuba. The work proposes a means of improving tutor training through three avenues: overcoming challenges, teaching assistant work and methodological work.
*Becoming a super preceptor: a practical guide to preceptorship in today's clinical climate.* [25]	The study proposes ways to apply realistic techniques to ensure that instruction is successful.
*Competencias docentes del Médico de Familia em el desempeño de la tutoría em la carrera de Medicina* *Article* [26]	The work verifies the absence of a system for selecting and training tutors. Moreover, the authors criticize a lack of suitable scenarios for teaching due to material difficulties. It is observed that inadequate teaching-service relationships have resulted in improvisation and a lack of preceptor motivation. The work calls for the use of skills that can improve pedagogical management among family doctors who serve as tutors.

^a^*SUS* Brazil’s Unified Health System

^b^*DCNs* National Curricular Guidelines

^c^*SMS* Health Department of Rio de Janeiro

^d^*IES* Higher Education Institutions

## Discussion

Our results show that issues related to instructor qualifications are recognized among teachers and students. Thus, means of improving qualifications for this profession should be explored, as noted by Demarzo (2011) [27].

Our findings reveal a variety of perceptions of and proposed approaches to (ideal) training in health care and, more specifically, in the PC and BFHU practices used in medical schools. It is noteworthy, therefore, that our study is fundamentally perceptual, that is, based on “impressions” or “apprehension through the senses”. These perceptions were assessed at the workshop meetings held at the BFHU in the second phase and are available in Additional file 1: Appendix F Tables 01 to 27. In this regard, Bollela et al. (2010), in their book “Internato Baseado em Competências” p.6, argue that competencies must be based on the following 4 essential aspects emphasized during instruction [28]:

### Cognitive functions

acquisition and use of knowledge to solve real-life problems;

### Integrative functions

use of biometric and psychosocial data to elaborate on clinical reasoning;

### Relationship functions

effective communication with patients, patients’ families and members of health care teams;

### Affective and moral functions

availability, patience, tolerance, and respect and the ability to use these attributes judiciously and humanely.

In this study, we prioritized "cognitive functions" and, more specifically, the "knowledge acquisition" that PC instructors may exhibit and/or perfect with undergraduates under their supervision.

The proposal to use electronic portals is consistent with the prioritization of cognitive functions and with the use of the electronic SBE portal of the Ministry of Health (MS) prepared in partnership with the Coordination for the Improvement of Higher Education Personnel initiative and the Ministry of Education and Culture (Capes/MEC). The portal allows one to navigate twelve health care databases in addition to a digital library of several scientific publications [12, 13, 22]. Other digital libraries include Elsevier Publishing House’s "evolution" portal and the BVS portal, which includes systematic reviews from Cochrane, the US National Library of Medicine, the National Institutes of Health, and PubMed. Finally, telemedicine and telehealth resources have been used and analyzed for their utility in collaborative research, tele-education and teleassistance through the National Telehealth Project of the Ministry of Health, which has conducted teleconference classes and distance learning courses.

However, a key question remains. How can we encourage students with Internet access to use these resources?

In Brazil, most BFHUs are located in urban areas, and there are four to five times more BFHUs in urban than in rural regions, mainly on the peripheries of large cities. Professionals working there as preceptors must carry out planning and activities in their units based on the diagnosis of the most frequent diseases in that community. It is also notable that the curriculum of the medical schools mentioned in this study, which is oriented toward work in BFHUs, emphasizes disciplines that seek to understand intimately the realities of the communities served (community-based clinical preceptories). Among these, we highlight “Family health”, “Collective health”; “Introduction to anthropology”; “Medical psychology”; “Integral medicine for children and adults” and “Indigenous health”. On the other hand, Starfield (2002) emphasizes that PC can be effective in meeting most of the demands of an organized and efficient health system. In this way, arousing the interest of students in using electronic tools in BFHUs, focusing in particular on the use of electronic portals, such as the electronic SBE portal of the Ministry of Health (MS) presented in our study, can be situated in the exact context of their effective, actual use, as pointed out in one of the practical questions in Table 2: How can we use pumpkin seeds? [6, 29, 30]

International surveys using internet resources show similar results about training with EBM criteria and the effectiveness of primary health care services. For example, in Hangzhou, China, in-person EBM training of health professionals was more effective than self-delivered EBM training for the management of arterial hypertension in the community. In addition, the United States Agency for Research and Quality in Health (AHRQ) invested in a PC research project "EvidenceNOW", which uses teleconsulting ) such as health information technology support), local learning collaboration and electronic consultation with experts to increase evidence-based practice in cardiovascular care in more than 1,500 care clinics [31, 32]

Thus, the use of well-founded electronic resources may be associated with higher levels of dissemination of information and knowledge in the academic environment, even across countries and cultures.

In sum, BFHU teaching/learning can significantly improve the cognitive outcomes in discussions of clinical issues when Internet resources such as electronic portals, virtual and digital libraries, and telemedicine and telehealth services are used.

## Conclusion

While we do not have an ideal model for PC instructor training, we recommend the use of the SBE Portal as a means to improve instructors’ cognitive functions and to assist BFHU professionals in their daily tasks. We emphasize the need for interaction among doctors, students and health professionals as a way to improve PC. In this way, we can contribute to their training and encourage good clinical and PC practices among medical students and medical school graduates, which will prove indispensable to the Brazilian population and populations worldwide.

## Supplementary Information

**Additional file 1.**

## Data Availability

The datasets used and/or analyzed in the current study are available from the corresponding author on reasonable request.

## Abbreviations

AHRQ: Agency for Healthcare Research and Quality
AMI: Acute Myocardial Infarction
BHFU: Basic Family Health Unit
BIREME: Latin American and Caribbean Center on Health Sciences Information
BMJ: British Medical Journal
BVS: Virtual Health Library
CAPES: Coordination for the Improvement of Higher Education Personnel
EBM: Evidence-Based Medicine
MEC: Ministry of Education and Culture
MS: Ministry of Health
OPAS: Pan American Health Organization
PC: Primary Care
RJ: Rio de Janeiro
SBE: Evidence-Based Health
TCLE: Terms of Free and Informed Consent
UEL: State University of Londrina
UFF: Federal Fluminense University
UFT: Federal University of Tocantins
UFV: Federal University of Viçosa
UNIFESO: University Center of Serra dos Órgãos
WFME: World Federation of Medical Education
WHO: World Health Organization
