# Supplementary material for: Use of an evidence-based health portal to improve teaching and learning in primary care: a mixed methods evaluation
Source: BMC Med Educ. 2021 May 24;21:296. doi: 10.1186/s12909-021-02686-9 (PMC8142472; doi:10.1186/s12909-021-02686-9)
Supplement: Supplementary file 1 — Additional file 1. [file 12909_2021_2686_MOESM1_ESM.docx]

**APPENDIXES**

**APPENDIX A**

**QUESTIONNAIRE FOR GRADUATE STUDENTS AND INTERNS AT INSTITUTIONS WITHOUT AN ACTIVE TEACHING-LEARNING METHODOLOGY**

**IDENTIFICATION:**

A) Institution: ___________________B) Gender: male ( ) female ( )

C) Date of birth: _________________

D) Course period: Student ( ) Which period? ____ Internship student ( )

**QUESTIONNAIRE:**

Provide ONE answer to each question except for questions 10 and 11. If you do not agree with any of the answers presented, please provide an answer after "other."

**01** - Why did you choose to study medicine?

( ) Family pressure

( ) Status and/or economic considerations

( ) Scientific interest

( ) Vocational interest

( ) Other____________________________________

**02** - Why did you to choose to study medicine at this institution?

( ) Public status

( ) Proximity

( ) Scientific interest and the opportunity to publish scientific works

( ) Institution reputation

( ) It was the only one I was accepted to

( ) Other____________________________________

**03** - How do you like the curriculum offered at your medical school?

( ) Very interesting

( ) Interesting

( ) Uninteresting

( ) I cannot provide an informed opinion

( ) Other____________________________________

**04** - Have you heard about active teaching-learning methodologies or their use at any medical school?

( ) Yes

( ) No

( ) Other____________________________________

**05** - If you answered "YES" or "other" with some knowledge about this teaching-learning mode, do you believe that the use of active teaching-learning methodologies presents any advantages over the curriculum offered at your school?

( ) Yes

( ) No

( ) Other____________________________________

**06** - If you answered "Yes" or "other" with some knowledge about this teaching-learning mode, what is your opinion about this mode? Otherwise, skip this question.

( ) My institution should incorporate this approach into its curriculum

( ) I believe that its implementation would be very difficult

( ) I prefer the current curriculum

( ) I have no opinion

( ) Other____________________________________

**07** - How have you found field activities in Primary Care (PC) or Basic Family Health Unit (BHFU) offered through your university education?

( ) Very interesting

( ) Interesting

( ) Uninteresting

( ) I cannot provide an informed opinion

( ) Other____________________________________

**08** - What would you maintain from this practice?

 ( ) The number of units

 ( ) The number of students enrolled in each unit

 ( ) Group activities with the community

 ( ) Home visits

( ) Field work or research

( ) Preceptors

( ) Participation in or the completion of Health Unit consultations

( ) Physical space offered by the Health Unit

 ( ) Other____________________________________

**09** - What would you change about this practice?

 ( ) The number of Unit participants: decrease ( ) increase ( )

 ( ) The number of students entering each unit: decrease ( ) increase ( )

 ( ) Group activities with the community

 ( ) Home visits

( ) Field work or research

( ) The preceptor

 ( ) The participation or the accomplishment of calls in the Unit: would decrease ( ) or increase ( )

 ( ) The time of this activity or the time that is spent in the Unit: would decrease ( ) or increase ( )

( ) The physical space offered by the Health Unit

 ( ) Other____________________________________

**10 -** How would you improve teaching and learning outcomes of PC or BHFU field activities? Provide answers of 1 to 10 with 1 denoting high priority and 10 denoting low priority. If you provide an answer after "other," provide a number as well.

( ) Local preceptor training

( ) Increasing the availability of local tutors for students

( ) Providing an preceptor external to the Unit

( ) Presence of a monitor or senior (more experienced) student at the Unit

( ) Use of resources such as commercial films

( ) Use of resources such as theatrical resources

( ) Use of resources such as videos

( ) Use of active teaching-learning methodologies

( ) Encouraging more work or field research

( ) Encouraging interaction with the community in the assigned area to facilitate learning

( ) Other ____________________________________

**11 –** What is the main problem facing teaching and learning in PC or BHFU field activities? Provide answers of 1 to 10 with 1 denoting the most important problem and 10 denoting the least important problem. If you provide an answer after "other,” number it as well.

( ) A lack of local preceptor training

( ) A lack of access to local preceptors for students

( ) A lack of preceptors external to units

( ) An absence of monitors or senior (more experienced) students from the unit

( ) A lack of commercial film resources

( ) A lack of theatrical resources

( ) A lack of video resources

( ) A lack of use of active teaching-learning methodologies

( ) Limited improvement of work or field research

( ) A lack of interaction with the community that could facilitate learning

( ) Other

**12 -** What effect is participation in PC/BHFU field activities with this institution having on your professional trajectory?

( ) Too much influence

( ) Some influence

( ) No influence

( ) I cannot provide an informed opinion

( ) Other____________________________________

**13 –** Have PC/BHFU Care field activities promoted by this institution had any influence on your professional trajectory?

( ) It increased my interest in family or general medicine

( ) It reinforced my interest in becoming a family physician or general practitioner

( ) It reinforced my lack of interest in becoming a family physician or general practitioner

( ) It inspired an interest in studying other areas/specialties. Which one?____________________________

( ) It reinforced my interest in studying other areas/specialties. ( ) Which one?____________________________

( ) Other____________________________________________

**14 –** Have PC or BHFU field activities promoted by this institution encouraged you to practice Family Medicine or General Medicine?

( ) Yes. It has had a considerable influence

( ) Yes. It has had some influence

( ) No. It has had no influence

( ) I cannot provide an informed opinion

( ) Other____________________________________

Data on the socioeconomic profiles of participants of this research are requested below: EVIDENCE-BASED FAMILY HEALTH: FROM CURRENT PRACTICE TO NEW TEACHING-LEARNING PROPOSALS AT MEDICAL SCHOOLS.

Age: _____ years;

Marital status: __________________________;

In your opinion, what is your ethnic background? Black Asian Caucasian Other______________________

Do you live with family members? No:___ Yes:___

Living situation: Own house/apartment:____ Rent house/apartment: ____ Other: ____

Do you have another college degree? No ____ Yes____

If so, what is it in? _______________________________

Do you have your own income? No Yes – What is your annual income? R$_______________;

Considering your income (if any) and that of any parents or guardians living with you, what is your family income?

( ) Up to five times the minimum wage (up to R$ 3,110.00)

( ) More than 5 to up to 10 times the minimum wage (R$ 3,110.00 to R$ 6,220.00)

( ) More than 10 to up to 15 times the minimum wage (R$ 6,220.00 to R$ 9,330.00)

( ) More than 15 to up to 20 times the minimum wage (more than R$ 9,330.00 to R$ 12,440.00)

( ) More than 20 times the minimum wage (more than R$ 12,440.00)

Father’s education:

( ) No education ( ) 1^st^ Degree ( ) 2^nd^ Degree ( ) 3^rd^ Degree ( ) Post-Graduate

Mother’s education:

( ) No education ( ) 1^st^ Degree ( ) 2^nd^ Degree ( ) 3^rd^ Degree ( ) Post-Graduate

Do you have a computer at home? No ( ) Yes ( )

Do you have access to the Internet? No ( ) Yes ( )

Do you own a car? No ( ) Yes ( )

Does your family own a car? No ( ) Yes ( )

In total, including textbooks, how many books do you own?

( ) Up to 50 ( ) 51 to 100 ( ) 101 to 200 ( ) 201 to 500 ( ) 501 to 1,000 ( ) more than 1,000

**APPENDIX B**

**QUESTIONNAIRE FOR GRADUATE STUDENTS AND INTERNS SUBJECTED TO THE ACTIVE TEACHING-LEARNING METHODOLOGY**

**IDENTIFICATION:**

A) Institution: ___________________

B) Gender: male ( ) female ( )

C) Date of birth: ______________________

D) Period: Student ( ) Which period? ____Internship student ( )

**QUESTIONNAIRE:**

Provide ONE answer to each question except for questions 11 and 12. If you do not agree with any answer provided, please provide an answer after "other.”

**01 -** Why did you choose to study medicine?

( ) Family pressure

( ) Status and/or economic considerations

( ) Scientific interest

( ) Vocational interest

( ) Other____________________________________

**02 –** Why did you choose to study medicine at this institution?

( ) Public status

( ) Proximity

( ) Scientific interest and the opportunity to publish scientific works

( ) Institution reputation

( ) It was the only one I was accepted to

( ) Other____________________________________

**03 -** If your institution has adopted active teaching/learning methodologies in your curriculum, how have you found these methodologies in medical education?

( ) Very interesting

( ) Interesting

( ) Uninteresting

( ) I cannot provide an informed opinion

( ) Other ____________________________________

**04 -** If you answered "uninteresting," "I cannot provide an informed opinion" or "other" with a negative response, justify this response; if your impression was positive, skip this question:

( ) Lectures are more effective

( ) I learn more when teachers provide lessons

( ) The new method is disorganized

( ) I cannot learn from the new method

( ) Other____________________________________

**05 -** If your institution has adopted active methodologies of teaching/learning, what are its advantages over the traditional method of teaching? If you have not enjoyed active methodologies, do not answer this question.

( ) Research autonomy

( ) Time to study

( ) Integrated knowledge

( ) More dynamic learning methods

( ) Other____________________________________

**06 -** What would you maintain from active teaching/learning methodologies?

( ) The number of students engaged in tutoring

( ) The tutor or preceptor

( ) The problems studied

( ) The time dedicated to tutoring

( ) Evaluations

( ) Other_____________________________________

**07 -** How would you change active teaching/learning methodologies?

( ) Increase the number of students engaged in tutoring

( ) Decrease the number of students engaged in tutoring

( ) The tutor or preceptor

( ) Increase time dedicated to tutoring

( ) Decrease time dedicated to tutoring

( ) Evaluations

( ) Problems studied

( ) Other____________________________________

**08 -** How have you found Primary Care (PC) or Basic Family Health Unit (BHFU) activities?

( ) Very interesting

( ) Interesting

( ) Uninteresting

( ) I cannot provide an informed opinion

( ) Other____________________________________

**09 -** What would you maintain from this practice?

 ( ) The number of students enrolled

 ( ) Group activities with the community

 ( ) Home visits

( ) Field work or research

( ) The preceptor

( ) Participation in Health Unit consultations

( ) The physical space offered by the Health Unit

 ( ) Other____________________________________

**10 -** What would you change about this practice?

 ( ) The number of Unit attendees: decrease ( ) or increase ( )

 ( ) The number of students entering the Unit: decrease ( ) or increase ( )

 ( ) Group activities with the community

 ( ) Home visits

( ) Field work or research

( ) The preceptor

 ( ) Participation in Unit calls: decrease ( ) increase ( )

 ( ) Time spent at the Unit: decrease ( ) or increase ( )

( ) The physical space offered by the Health Unit

( ) Other____________________________________

**11 -** How would you recommend improving PC or BHFU teaching and learning? Provide answers of 1 to 10 with 1 denoting the highest priority and 10 denoting the lowest priority. If you provide an answer after "other,” number it as well.

( ) Local preceptor training

( ) Increasing student access to local tutors

( ) Providing an preceptor external to the unit

( ) Providing a monitor or senior (more experienced) student with the unit

( ) Use of resources such as commercial films

( ) Use of tools such as theatrical resources

( ) Use of resources such as videos

( ) Use of active teaching-learning methodologies

( ) Encouraging work or field research

( ) Encouraging interaction with the community to facilitate learning

( ) Other____________________________________

**12 –** What is the main problem facing PC or BHFU teaching and learning presents? Provide answers of 1 to 10 with 1 denoting the most important problem and 10 denoting the least important problem. If you provide an answer after "other,” number it as well.

( ) Lack of local preceptor training

( ) Lack of local preceptors to help students

( ) Lack of preceptors external to the unit

( ) Absence of a monitor or senior (more experienced) student with the unit

( ) Lack of resources such as commercial films

( ) Lack of resources such as theatrical resources

( ) Lack of resources such as videos

( ) Lacking use of active teaching-learning methodologies

( ) Lacking work or field research

( ) Lack of interaction with the community to facilitate learning

( ) Other____________________________________

**13 –** How much of an influence has participation in PC/BHFU activities at this institution had on your professional trajectory?

( ) Too much influence

( ) Some influence

( ) No influence

( ) I cannot provide an informed opinion

( ) Other____________________________________

**14 -** How much of an influence has the use of the active teaching-learning methodologies at this institution had in your professional trajectory? If your institution has NOT adopted such methods in your medical curriculum, skip this question.

( ) Too much influence

( ) Some influence

( ) No influence

( ) I cannot provide an informed opinion

( ) Other____________________________________

**15 –** How has the new curricular model (active methodology and use in BHFUs) adopted by this Institution affected your professional trajectory? If your institution has NOT adopted this approach in your medical curriculum, skip this question.

 ( ) It reinforced my interest in studying Family or General Medicine

( ) It made me interested in studying other areas/specialties

( ) It inspired a sense of autonomy in my professional life

( ) It made me feel more able to practice medicine

( ) I learned how to better manage my study time

( ) Other____________________________________

**16 -** Have new practice scenarios such as PC or BHFU promoted by this institution affected your professional trajectory?

( ) It increased my interest in becoming a Family physician or general practitioner

( ) It reinforced my interest in becoming a Family Physician or general practitioner

( ) It reinforced my lack of interest becoming a Family Physician or general practitioner

( ) It made me interested in studying other areas/specialties Which ones?____________________________

( ) It reinforced my interest in studying other areas/specialties Which ones?____________________________

( ) Other____________________________________________

**17 -** Have new practice scenarios such as PC or BHFU promoted by this institution inclined you to practice Family or General Medicine?

( ) Yes

( ) Yes

( ) No, no influence

( ) I cannot provide an informed opinion

( ) Other____________________________________

Data on the socioeconomic profile of the participants of this research are requested below. EVIDENCE-BASED FAMILY HEALTH: FROM CURRENT PRACTICE TO NEW TEACHING-LEARNING PROPOSALS AT MEDICAL SCHOOLS

Age, years;

Marital status: __________________________;

In your opinion, what is your ethnic background: Black Asian Caucasian Other______________________

Do you live with family members? No Yes

Housing situation: Own house/apartment Rent house/apartment Other

Do you have another college degree? No Yes – What is it in? __________________________________________;

Have you worked in or studied another field: No Yes - Which one? __________________________________________;

Do you have your own income? No Yes - How much do you earn? R$______________________;

Given your income (if any) and those of any parents or guardians living with you, what is your family’s annual income?

( ) Up to five times the minimum wage (up to R$ 3,110.00)

( ) More than 5 to up to 10 times the minimum wage (R$ 3,110.00 to R$ 6,220.00)

( ) More than 10 to up to 15 times the minimum wage (R$ 6,220.00 to R$ 9,330.00)

( ) More than 15 to up to 20 times the minimum wage (more than R$ 9,330.00 to R$ 12,440.00)

( ) More than 20 times the minimum wage (more than R$ 12,440.00)

Father’s education:

( ) No education ( ) 1^st^ Degree ( ) ^2nd^ Degree ( ) 3^rd^ Degree ( ) Post-Graduate

Mother’s education:

( ) No education ( ) 1^st^ Degree ( ) ^2nd^ Degree ( ) 3^rd^ Degree ( ) Post-Graduate

Do you have a computer at home? No ( ) or Yes ( )

Do you have access to the Internet? No ( ) or Yes ( )

Do you own a car? No ( ) or Yes ( )

Does your family own a car? No ( ) or Yes ( )

In total, including textbooks, how many books do you own?

( ) Up to 50 ( ) 51 to 100 ( ) 101 to 200 ( ) 201 to 500 ( ) 501 to 1,000 ( ) more than 1,000

**APPENDIX C**

**QUESTIONNAIRE FOR PRECEPTORS**

**EVIDENCE-BASED FAMILY HEALTH: FROM CURRENT PRACTICE TO NEW TEACHING-LEARNING PROPOSALS AT MEDICAL SCHOOLS**

Role: ( ) Doctor ( ) Nurse ( ) Community Agent ( ) Institution Teacher

( ) Other: ___________________

Do you have a specialization, or have you completed residency? ( ) Yes ( ) No

Which one or which ones?

Do you have any specific training in ​​Family and Community Medicine?

**QUESTIONS:**

**1)** How do you define your role as a preceptor?

___________________________________________________________________________________________________________________________________________________________________________________________________________________________________________________________________________________________________________________________

**2)** What are your views on students entering Primary Care (PC) or Basic Family Health Unit (BHFU)

____________________________________________________________________________________________________________________________________________________________________________________________________________________________________________________________

**3)** What are your views on your instruction of PC or BHFUs with medical students?

_________________________________________________________________________________________________________________________________________________________________________________________________________________________________________________________________________________________________________________________________________________________________________________________

**4)** Are you satisfied with the teaching and learning that occurs in PC or BHFUs? How would you recommend improving teaching and learning in these areas?

____________________________________________________________________________________________________________________________________________________________________________________________________________________________________________________________

**APPENDIX D1**

**Student - Questionnaire (nº 1)**

Dear student, complete the following questionnaire (nº 1) by selecting a single answer to each statement:

Period: _____ Basic Unit: __________________________

| **Statements** | **DT** | **DP** | **NC ND** | **CP** | **CT** |
| --- | --- | --- | --- | --- | --- |
| 1) Teaching and learning in Primary Care (PC) are inadequate |  |  |  |  |  |
| 2) I have knowledge of medical research portals |  |  |  |  |  |
| 3) I have knowledge of Telemedicine and Telehealth resources |  |  |  |  |  |
| 4) I have knowledge of digital libraries |  |  |  |  |  |
| 5) My training in online tools such as research sites, digital libraries, Telemedicine and Telehealth has been important |  |  |  |  |  |
| 6) My training in online tools such as research sites, digital libraries, Telemedicine and Telehealth has been more important than my use of printed books and my institution’s library |  |  |  |  |  |
| 7) I have knowledge of Evidence-Based Medicine (EBM) |  |  |  |  |  |
| 8) I apply EBM in PC |  |  |  |  |  |
| 9) I apply EBM in other practice settings |  |  |  |  |  |
| 10) The application of EBM can improve my knowledge base |  |  |  |  |  |
| 11) The application of EBM can improve my performance in PC |  |  |  |  |  |
| 12) My teachers or preceptors have knowledge of EBM |  |  |  |  |  |
| 13) I discuss medical practices based on EBM with my teachers or preceptors |  |  |  |  |  |

DT: I completely disagree

DP: I partially disagree

NC/ND: I do not agree or disagree

CP: I partially agree

CT: I completely agree

Question: What are your views on the possibility of applying online tools such as research sites, digital libraries, Telemedicine and Telehealth? _____________________________________________________________________________________________________________________________________________________________________________________________

**APPENDIX D2**

**Student - Questionnaire (nº 2)**

Complete the following questionnaire (nº 2) by selecting a single response to each statement:

Period: _____ Basic Unit: __________________________

| **Statements** | **DT** | **DP** | **NC ND** | **CP** | **CT** |
| --- | --- | --- | --- | --- | --- |
| 1) Primary Care teaching and learning are inadequate |  |  |  |  |  |
| 2) I have knowledge of medical research portals |  |  |  |  |  |
| 3) I have knowledge of Telemedicine and Telehealth resources |  |  |  |  |  |
| 4) I have knowledge of digital libraries |  |  |  |  |  |
| 5) My training in online tools such as research sites, digital libraries, Telemedicine and Telehealth has been important |  |  |  |  |  |
| 6) My training in online tools such as research sites, digital libraries, Telemedicine and Telehealth has been more important than my use of printed books and my institution’s library |  |  |  |  |  |
| 7) I have knowledge of Evidence-Based Medicine (EBM) |  |  |  |  |  |
| 8) I apply EBM in Primary Care (PC) |  |  |  |  |  |
| 9) I apply EBM in other practice settings |  |  |  |  |  |
| 10) The application of EBM can improve my knowledge base |  |  |  |  |  |
| 11) The application of EBM can improve my performance in PC |  |  |  |  |  |
| 12) My teachers or preceptors have knowledge of EBM |  |  |  |  |  |
| 13) I discuss medical practices based on EBM with my teachers or preceptors |  |  |  |  |  |

DT: I completely disagree

DP: I partially disagree

NC/ND: I do not agree or disagree

CP: I partially agree

CT: I completely agree

Question: What are your views on the possibility of applying online tools such as research sites, digital libraries, Telemedicine and Telehealth? _____________________________________________________________________________________________________________________________________________________________________________________________

**APPENDIX E1**

**Preceptor - Questionnaire (nº 1)**

Complete the following questionnaire (nº 1) by selecting a single response to each statement:

Period: _____ Basic Unit: __________________________

| **Statements** | **DT** | **DP** | **NC ND** | **CP** | **CT** |
| --- | --- | --- | --- | --- | --- |
| 1) My training as an preceptor is inadequate |  |  |  |  |  |
| 2) I have knowledge of medical research portals |  |  |  |  |  |
| 3 I have knowledge of Telemedicine and Telehealth resources |  |  |  |  |  |
| 4) I have knowledge of digital libraries |  |  |  |  |  |
| 5) My training in online tools such as research sites, digital libraries, Telemedicine has been Telehealth are important |  |  |  |  |  |
| 6) My training in online tools such as research sites, digital libraries, Telemedicine and Telehealth has been more important than my use of printed books and my institution’s library |  |  |  |  |  |
| 7 ) I have knowledge of Evidence-Based Medicine (EBM) |  |  |  |  |  |
| 8) I apply MBE in my practice and in discussions on Primary Care (PC) |  |  |  |  |  |
| 9) The application of EBM can improve my knowledge base |  |  |  |  |  |
| 10) The application of EBM can improve my performance in PC |  |  |  |  |  |
| 11) My students have knowledge of EBM |  |  |  |  |  |
| 12) I discuss EBM with my students |  |  |  |  |  |

DT: I completely disagree

DP: I partially disagree

NC/ND: I do not agree or disagree

CP: I partially agree

CT: I completely agree

Question: What are your views on the possibility of applying online tools such as research sites, digital libraries, Telemedicine and Telehealth? _____________________________________________________________________________________________________________________________________________________________________________________________

**APPENDIX E2**

**Preceptor - Questionnaire (nº 2)**

Complete the following questionnaire (nº 2) by selecting a single response to each statement:

Period: _____ Basic Unit: __________________________

| **Statements** | **DT** | **DP** | **NC ND** | **CP** | **CT** |
| --- | --- | --- | --- | --- | --- |
| 1) My training as an preceptor is inadequate |  |  |  |  |  |
| 2) I have knowledge of medical research portals |  |  |  |  |  |
| 3) I have knowledge of digital libraries |  |  |  |  |  |
| 4) My training in online tools such as research sites, digital libraries, Telemedicine and Telehealth has been important |  |  |  |  |  |
| 5) My training in online tools such as research sites, digital libraries, Telemedicine and Telehealth has been more important than my use of printed books and my institution’s library |  |  |  |  |  |
| 6) I have knowledge of Evidence-Based Medicine (EBM) |  |  |  |  |  |
| 7) I apply EBM in my practice and in discussions on Primary Care (PC) |  |  |  |  |  |
| 8) The application of EBM can improve my knowledge base |  |  |  |  |  |
| 9) The application of EBM can improve my performance in PC |  |  |  |  |  |
| 10) My students have knowledge of EBM |  |  |  |  |  |
| 11) I discuss EBM with my students |  |  |  |  |  |

DT: I completely disagree

DP: I partially disagree

NC/ND: I do not agree or disagree

CP: I partially agree

CT: I completely agree

Question: What are your views on the possibility of applying online tools such as research sites, digital libraries, Telemedicine and Telehealth? ___________________________________________________________________________________________________________________________________________________________________________________________________

**APPENDIXES F**

**Tables from 01 to 27**

| **Table1: The first meeting: our first Workshop...** | |
| --- | --- |
| **Location: Araras Family Health Basic Unit** | |
| **Date and Suggested Activity** | **Results** |
| **09/07/2012**  **a) Presentation and purpose of the thesis**  **b) The problem pointed out: Training and availability of the preceptor**  **c) Presentation and discussion about the search tools and the Evidence-Based Health Portal (SBE) and Telehealth:**[**http://aplicacao.periodicos.saude.gov.br/index.php?view=principal**](http://aplicacao.periodicos.saude.gov.br/index.php?view=principal)  **d) Possible choice of subjects for study** | In the Unit, there are four internship students of the 9^th^ period. The workshop was held with two internship students and the presence of the nurse and the preceptor of the Family Health Basic Unit (BHFU) to identify two conditions:  1) Regarding teaching and learning in BHFU: What would be the biggest problem?  2) What would you suggest to improve teaching and learning at BHFU?  It was identified the lack of development of themes that were not fully developed or that were deficient in the Tutoring Sessions in the Medical School Campus in the Alto district as pharmacology and anatomy. The internship student did not specifically report complaints to the BHFU, but they would like to contextualize these issues in the care in the Unit.  Suggestion: Indication and Pharmacodynamics of the main antihypertensives.  It was also discussed the accessibility to treatment (free of charge and proximity to the pharmacy) and its adherence |

| **Table 2: The holidays...** | |
| --- | --- |
| **Location: Araras Family Health Basic Unit** | |
| **Date and Suggested Activity** | **Results** |
| **16/07/2012 to 30/07/2012** | **Holidays in BHFU. No students** |

| **Table 3: Reflections on Evidence-Based Medicine...** | | |
| --- | --- | --- |
| **Location: Araras Family Health Basic Unit** | | |
| **Date and Suggested Activity** | **Results** | |
| **20/08/2012**  **Rediscussion on the themes proposed in the workshop of June: pharmacodynamics of antihypertensives** | Workshop with the participation of three internist and the preceptor. Perception of Evidence-Based Medicine (EBM). Presentation by slides in notebook on EBM. Perception of how MBE could be applied to the pharmacological study of antihypertensive drugs and daily clinical topics of a Family Health Basic Unit (BHFU).  The students were not participatory and apparently did not show much interest in the topic offered. The preceptor was quite distressed by the demand for attendance at the Unit although she found the subject of EBM quite interesting | |
| **Table 4: Missing physician...** | | |
| **Location: Araras Family Health Basic Unit** | | |
| **Date and Suggested Activity** | | **Results** |
| **27/08/2012**  **Whithout activity** | | Internist left earlier. Health problems with the mother of the doctor |

| **Table 5: The resistance to Evidence-Based Medicine speech...** | |
| --- | --- |
| **Location: Araras Family Health Basic Unit** | |
| **Date and Suggested Activity** | **Results** |
| **03/09/2012**  **Discussion on Evidence-Based Medicine (EBM)** | Discuss again on the validity of MBE in clinical decision making (with 04 students, since the preceptor was busy with the Unit's demand).  Resistance to EBM principles. For students this opinion is impractical in everyday life.  Arguing that the preceptor would not change his practical perception and that nothing would help the MBE in the daily life.  They would not like to turn these workshops into tutorial sessions, for which they had already had several classes on Campus and that would already be saturated.  Tense receptivity. |

| **Table 6: Finally, the discussion applies to clinical practice...** | |
| --- | --- |
| **Location: Araras Family Health Basic Unit** | |
| **Date and Suggested Activity** | **Results** |
| **10/09/2012**  **Discussion of the conducts on cystitis (bladder infection)** | Discussion of the conduct with thiazide diuretic. Comparison on the validity of the studies presented in Consensus comparing them to Evidence-Based Medicine (MBE) with the best practice database. Doubts in the conduct of the preceptor on cystitis in men (03 or 07 days of antibiotic therapy?).  The treatment with Sulfa and Trimetropim for simple cystitis was verified in this portal, which confirmed the behavior of the preceptor in many cases of this type of affection, that is, the option for the antibiotic therapy with Sulfa and trimetropim for simple cystitis.  It was also verified that there was no similarity between the conduct for initial treatment of SAH of the Guidelines of the Brazilian Society of Cardiology that instituted treatment with monotherapy from beta blockers or even calcium channel antagonists in addition to diuretics in relation to the database best practice of the Evidence-Based Health Portal (SBE) that guided the use of diuretics as monotherapy for this initial treatment. The discussion on iron deficiency anemia was arranged for the next workshop. Interns participated and liked the Workshop as well as the governess |

| **Table 7: Internists do not go to the Unit...** | |
| --- | --- |
| **Location: Araras Family Health Basic Unit** | |
| **Date and Suggested Activity** | **Results** |
| **17/09/12**  **Whithout activity** | *Internists did not go to the Unit. They agreed during the week with the Health team for some demand of the Course of Medicine in the Campus* |

| **Table 8: And the new group does not come either....** | |
| --- | --- |
| **Location: Araras Family Health Basic Unit** | |
| **Date and Suggested Activity** | **Results** |
| **27/09/12**  **Whithout activity** | *Students of the fourth period did not come due to the need to study for evaluation that would be on September 29 in the Campus by agreement with the Health Unit.*  *Unfortunately, we opted for the abandonment of the application of the Thesis in this Unit.* |

| **Table 9: Now, let us go to the Campus...** | |
| --- | --- |
| **Location: Campus of UNIFESO** | |
| **Date and Suggested Activity** | **Results** |
| **Months of October and November 2012**  **Participation in weekly meetings of the preceptors of the Basic Units of Family Health** | Participation in weekly meetings - which were already carried out by the teams - at the UNIFESO campus - in an attempt to offer the application of the Thesis based on the autonomy of the Unit's own timetable with its students and with the possibility of obtaining its application in several Units simultaneously. There were 06 meetings.  It is worth mentioning that this time we have perfected ourselves. All contact with the teams was more organized. Presentations and distribution of tutorials for publishing and digital libraries from free electronic portals were made, as well as the systematic for registration of Telehealth and Telehealth portals of the "National Telehealth Project" (Telehealth Brazil Networks). These tutorials were available in envelopes distributed for each Unit (14 Units), containing tables for date annotations, activities carried out (Study Topics) and impressions of the tutor and student in the Unit. This activity was proposed for the end of the year 2012 until the end of the year 2013. See appendices of the KIT  At this stage, despite approval by the Ethics Committee of FIOCRUZ and UNIFESO, we also request the authorization of the Medicine Coordination for the application of the thesis to the BHFU See Appendixes.  Some Units were willing to apply the study in the month of November, but they already claimed that they would be at the end of the school year and that there would be no continuity of the studies by the students. They opted to start in January 2013. |

| **Table 10: Waiting for the holidays...** | |
| --- | --- |
| **Location: Campus of UNIFESO** | |
| **Date and Suggested Activity** | **Results** |
| **December 2012**  **Whithout activity** | Recess of teachers/preceptors in Units and students |

| **Table 11: Waiting for the holidays...** | |
| --- | --- |
| **Location: Campus of UNIFESO** | |
| **Date and Suggested Activity** | **Results** |
| **January 2013**  **Whithout activity** | *We had already noticed at the end of the meetings in November that there would be participation or adherence of a few or even risk of not having any Unit in the application of the Thesis. Again, the allegation would be the lack of time and availability for participation and the realization of workshops and with the possibility of interfering in the work process of the Unit.*  *As the preceptor of the Vargem Grande Unit, located 15 km from the Center in a rural area of the municipality of Teresópolis, was available and interested, it was decided to carry out the Thesis Application integrally in this Unit, becoming our pilot unit from Of this month.* |

| **Table 12: Finally the reception and the beginning of fact...** | |
| --- | --- |
| **Location: Vargem Grande Family Health Basic Unit (rural Unit)** | |
| **Date and Suggested Activity** | **Results** |
| **24/01/13**  **Workshops always on Tuesday or Wednesday in an afternoon shift with the participation of the preceptor and the five interns crowded there**  **1^st^ Group of Interns ("run" every three months)**  **Application of the Pre-test with the Likert scale.**  **Use of the doctoral and preceptor login and password to access the SBE Portal** | Attending: Preceptor  Students: 05 Internists  Start of activity in the Unit/Presence of all Internal of the Ninth period Presentation of the Research Project. Application of the Pre-test with the Likert scale. MBE and Telehealth discussion  Welcome to the proposal to discuss clinical issues  • Themes chosen from consensus with the group and the meaning of the same for students and preceptor.  • Discussion of topics from very practical issues such as a TV show call. Elemental questions that include broader questions. It was decided to discuss for the next week:  • Is the prophylactic use of acetylsalicylic acid (Aspirin®) beneficial to heart disease patients?  • Is prophylactic use of Aspirin® beneficial to healthy people?  • Is physical activity beneficial to those who have had AMI?   - • And for those who did not? |

| **Table 13: Who needs Aspirin^®^?...** | |
| --- | --- |
| **Location: Vargem Grande Family Health Basic Unit (rural unit)** | |
| **Date and Suggested Activity** | **Results** |
| **31/01/13**  **- Discussion of the proposed themes** | Attending: Preceptor  Students: 05 Internists  All used the VHL portal  The preceptor sought to cross the terms exercise x physical x advantages and ended up selecting the article: Knowledge of adults on the role of physical activity in the prevention and treatment of diabetes and hypertension: a population-based study in southern Brazil  Cad. Saúde Pública vol.25 nº.3 Rio de Janeiro Mar. 2009 Available in:http://www.scielo.br/scielo.php?pid=S0102311X2009000300006&script=sci_arttext  *In this article, the preceptor verified that the knowledge of the population of Pelotas-RS on the role of physical activity in the treatment of diabetes and hypertension is greater in comparison to prevention and that, therefore, the importance of knowledge about prevention should be emphasized.*  *We verified that the students participated actively (although this was the first discussion and we could have had more doubts) having one of the Interns read the chapter regarding the pharmacological properties of aspirin in a book of Pharmacy, although it did not answer the question about the prophylactic use of Aspirin if it would be beneficial for heart patients and / or healthy people.*  *Another student verified in an article that the use of aspirin would reduce the risk of Acute Myocardial Infarction (AMI) by 35%. Pondered on the use of aspirin and the possibility of developing gastric diseases, stroke and digestive hemorrhage since this article dealt with this topic. The issue of physical activity is beneficial for those who have had or not had AMI was not answered by any of the students.*  *The remaining students were not able to access the site of the books “evolution”.*  **For pHD student:**  *1) Search the VHL portal for aspirin and infarction*  *Use of terms: ASPIRINA AND INFARTO*  *1. Separate: 758 complete texts*  *2. Separation: Subject: Aspirin -277- times*  *3. New separation: Subject: IAM - Aspirin AND IAM: 167 times*  ***Selected article:***  *Effects of aspirin and trapidil on cardiovascular events after acute myocardial infarction / Effects of aspirin and trapidil on cardiovascular events after acute myocardial infarction*  *Source: Rev Bras Med; 58 (11): 867-874, nov. 2001. tab, graf*  ***CONCLUSION:*** *Low-dose aspirin effectively prevented AMI relapse in post-infarction patients after thrombolysis or coronary angioplasty when used for long-term use.*  *We chose to study from the database "Best Practice" on the portal "evidence-based health" with a search for "Clinical evidences" and later "Systematic reviews" and then "Cardiovascular disorders"*  *• Primary prevention of cardiovascular disorders: physical activity*  *The most interesting question in this type of search was the fact that the questions offered in this database (Best Practice) from systematic reviews are coherent with the questions formulated and chosen in the Unit itself. Below is a list of the clinical questions addressed in this review offered by the database "Best Practice" in relation to the primary prevention of cardiovascular disorders (CVD):* 1) Does counselling people to increase physical activity lead to increased physical activity in healthy people without existing CVD?  **Probably beneficial:**  Counselling people to increase physical activity versus no counselling: effects on level of physical activity  Counselling people to perform higher- versus lower-intensity exercise programmes: effects on level of physical activity  2) What are the health benefits of increasing physical activity in relation to cardiovascular outcomes in healthy people without existing CVD?  **Unknown Efficacy:**  Counselling people to increase physical activity versus no advice: effects on cardiovascular outcomes  Counselling people to perform higher- versus lower-intensity exercise programmes: effects on cardiovascular outcomes  **Impression:** It can be noticed that the database "Best Practice" already offers a systematic review of the clinical questions and that, therefore, starting from clinical evidences instead of searching the VHL portal in isolated articles seems to be more to the preceptor and above all to the BHFU students.  It was agreed for the next week the study of:  • Smoking and cardiovascular diseases  • Diet and CVD |

| **Table 14: Beware of salt! Cigarettes? No way...** | |
| --- | --- |
| **Location: Basic Health Unit of Vargem Grande** | |
| **Date and suggested activity** | **Results** |
| **05/02/13**  **Discussion of proposed topics** | Attending: Preceptor  Students: 04 Internists  Two interns studied together in the VHL crossing terms Food and cardiovascular diseases. They cited three articles:  1) Level of knowledge about cardiovascular risk factors in a community of Naguanagua, Venezuela/The level of knowledge regarding cardiovascular risk factors in people living in Naguanagua, Venezuela.  Source: Rev Salud Publica (Bogota); 13 (5): 759-771, Oct. 2011. ilus, tab.  Available: http://pesquisa.bvsalud.org/regional/resources/lil-625641  The article demonstrates a relationship between the high risk factors presented and the low knowledge about them in the communities of Venezuela.  2) Excess salt may cause cardiovascular diseases of 2011 (Did not cite the source)  They say that the WHO recommends the intake of <5.0 grams of sodium and that there are policies of the Brazilian Federal Government to reduce the supply of salt (sodium) in processed foods so that there is a reduction in the use of antihypertensives GOULART, Denise et al. Tabagismo em idosos. Rev. Bras. Geriatr. Gerontol. [online]. 2010, vol.13, n.2, pp. 313-320. ISSN 1809-9823  Available: at: http://revista.unati.uerj.br/scielo.php?script=sci_abstract&pid=S1809-98232010000200015&lng=en&nrm=iso&tlng=en  3) DASH Diet in Reducing Blood Pressure and Prevention of Stroke / DASH diet in reducing blood pressure and preventing stroke.  Source: Sci. Med; 22 (2) Apr-Jun. 2012.  Available: http://pesquisa.bvsalud.org/regional/resources/resources/lil-66132  "The available evidence suggests that changes in lifestyle, including the adoption of a DASH-like diet, are effective strategies for controlling hypertension and reducing cardiovascular events, such as stroke.  In relation to smoking these same two inmates used the term smoking only and opted for the article:  GOULART, Denise et al. Smoking in the elderly. Rev. Bras. Geriatr. Gerontol. [online]. 2010, vol.13, n.2, pp. 313-320. ISSN 1809-9823.  Perception that smoking accelerates aging and that smoking represents a source of risk and the main cause of numerous diseases and that cessation of smoking, at any age, reduces the risk of death and improves overall health.  The third student also searched the VHL by crossing the terms: diet and cardiovascular diseases did not search for smoking. He presented two articles:  1) Diet Variety is Protective Factor for High Systolic Blood Pressure  Arq. Bras. Cardiol. vol.98 no.4 São Paulo Apr. 2012 Epub Mar 15, 2012  Available: at: http://www.scielo.br/scielo.php?script=sci_arttext&pid=S0066-782X2012000400008&lng=en&nrm=iso  2) Eating habits and risk factors for atherosclerosis in students from Bento Gonçalves (RS) 2010  Arq. Bras. Cardiol. vol.95 no.2 São Paulo Aug. 2010 Epub July 09, 2010  Available: http://www.scielo.br/scielo.php?script=sci_arttext&pid=S0066782X2010001200005&lng=en&nrm=iso  A study of 600 students indicated that interventions are needed to promote changes in students' eating habits such as increased consumption of fruits, vegetables and legumes and increased levels of physical activity.  According to this internal Pubmed did not seem so good. O preceptor optou pela busca na BVS com os termos tabagismo and AMI.  Risk factors associated with acute myocardial infarction in the metropolitan region of São Paulo. A region developed in a developing country.  This study demonstrates that risk factors (such as lipid profile, hip waist measurement) are independently associated with AMI in the metropolitan region of São Paulo. Álvaro Avezum, Leopoldo Soares Piegas, Júlio César R. Pereira  São Paulo, SP  Available: http://www.scielo.br/pdf/abc/v84n3/a03v84n3.pdf  Research by doctoral student:  1) Research in "Best Practice"  It was decided to study from the database "Best Practice" in the portal "evidence-based health" with search in "Clinical evidence" and later "Systematic reviews" and then "Cardiovascular disorders" having been selected there:  • Primary prevention of CVD: diet  *What are the effects of dietary advice in generally healthy adults without existing CVD or increased CVD risk factors to improve cardiovascular outcomes (mortality, cardiovascular events, and cardiovascular risk factors)?*  *Probably beneficial*  *Advice to reduce sodium intake alone*  *Unknown Efficacy*  *Advice to increase fiber intake alone*  *Advice to increase fruit and vegetable intake alone*  *Advice to reduce and / or modify fat intake alone*  *2) VHL research using the terms smoking and IAM*  *Selected two articles:* a) Smoker's paradox' in young patients with acute myocardial infarction.  b) The smoker's paradox after successful fibrinolysis: reduced risk of reocclusion but no improved long-term cardiac outcome.  *Interesting articles that reflect the so-called paradoxical effect in which smoking seems to be associated with better clinical outcomes in young AMI patients, suggesting the existence of a "smoker's paradox".*  Printing: Again, the database "Best Practice" already offers a systematic review of clinical questions and, therefore, starting from this item with its clinical evidences instead of searching the VHL portal in isolated articles is more advantageous.  Scheduled: Application of Omega 3 and 6. What real benefits can come from using it?  Proposal of submission of the Project to the Scientific Initiation Project of the PICPE Institution until the end of February. |

| **Table 15: Let's study at Telehealth?...** | |
| --- | --- |
| **Location: Campus of UNIFESO - Library** | |
| **Date and suggested activity** | **Results** |
| **02/03/13**  **Discussion only with the preceptor about the SBE Portal and telehealth** | Discussion exceptionally scheduled due to the carnival holiday in the Central Library of UNIFESO.  Only the preceptor appeared.  We discussed the methodology employed - Porto SBE - and the registration in the UHEJ's Telessaude for the Evidence-Based Clinical Research Course  http://www.telessaude.uerj.br/site/  It was decided to reschedule for 03/03: Application of Omega 3 and 6. What real benefits can come from using it? |

| **Table 16: Do you want a fish fillet?** | |
| --- | --- |
| **Location: Basic Health Unit of Vargem Grande** | |
| **Date and suggested activity** | **Results** |
| **05/03/13**  **Discussion of proposed topics** | Attending: Preceptor  Students: 05 Internist  Omega 3 and 6. What real benefits can come from using it?  One of the inmates used the "ProQuest" database of Evidence Based Health  Checked term Omega 3. He justified that he would like to know the action of omega 3 in the body.  He entered the site of the American Heart Association and verified the recommendation to eat four times a week a fillet of fish for those who had previous heart disease -150 mg for each fillet of fish being cold water fish: Salmon and Tuna in addition to sardines and oil decreased triglycerides and increased HDL. He cited care with the contamination of heavy metals in fish meat. He studied another article regarding this type of food to improve CNS synapses and amelioration of bipolar disorder (Not cited Article).  Second internist:  Use of VHL: Used term Omega 3 (read the titles that were associated with omega 3): Omega 3 fatty acids and treatment of schizophrenia. Journal of Clinical Psychiatry 2010.  He found that nerve tissue has many lipids and that Omega 3 would help in cell signaling and in enzymatic and neuronal regulation. 500mg is ideal for normal individuals and for schizophrenics it should be offered 4 times more. Helps prevent more severe psychopathies (even under the use of anti-psychotics) Its adverse effects would be increased total cholesterol and bleeding time increased. However, there would be better adherence to treatment and better response.  Third Internist:  He studied about the presentation of free radicals although he did not bring any article; ended up little collaborating in the discussion.  Fourth internist: BVS: Terms Omega 3 and benefícios Nutrire Magazine  She verified that there is a good correlation between neonatal development and the use of Omega three by pregnant women: aspects related to their essentiality and supplementation.  Fifth internist:  VHL: Used Omega 3 and later prevention and omega 3.  Balance between omega 3 and 6 fatty acids in the inflammatory response in patients with cancer and cachexia. Revista Nutrição vol. 19 n5 from Campinas.  Supplementation would decrease proinflammatory oxytocin  Supplementation with fish oil with 2.2 grams for 3 weeks would exert Antineoplastic and anticaquetic activity.  For doctorate:  Use of the Evidence-Based Health Portal:  Selected database: "Dynamed": Use of the term: Omega-3-acid Ethyl Esters  Results:   - A-Depression alternative treatments Updated 2012 Dec 05 12:39:00 PM:omega-3 fatty acids may reduce depression (level 2 [mid-level] evidence) but evidence inconsistent - omega-3 fatty acids may be considered as augmentation to antidepressant therapy (APA Category III) (APA 2010 Nov) - omega-3 supplementation does not appear to reduce depressive symptoms in patients with major depressive episodes except possibly in patients without comorbid anxiety disorders (level 2 [mid-level] evidence) - addition of omega-3 acid ethyl esters are no more effective than placebo in patients with major depression and coronary heart disease taking sertraline (level 1 [likely reliable] evidence) - omega-3 fatty acids (Omacor capsule) 1 g/day does not reduce mortality or risk for cardiovascular events in patients with impaired glucose tolerance or diabetes and high risk for cardiovascular events (level 1 [likely reliable] evidence) - *In summary, this portal made the following assertions:* **Evidence summaries inconsistent for clinical outcomes:** - no evidence that dietary or supplementary omega-3 fatty acid intake alters risk of death, cardiovascular events, or cancer - omega-3 fatty acid supplementation does not appear effective in secondary prevention of cardiovascular disease (level 2 [mid-level] evidence) - advice to increase fish oil intake has uncertain effects on cardiac mortality in patients with heart disease (level 2 [mid-level] evidence) - omega-3 fatty acid supplementation may reduce risk of sudden cardiac death in patients with previous myocardial infarction, but may increase risk in patients with angina (level 2 [mid-level] evidence) - omega 3 fatty acid and vitamin B supplements in combination or alone not associated with decreased risk of cardiovascular events in patients with previous myocardial infarction or stroke (level 2 [mid-level] evidence) - omega-3 fatty acid supplementation associated with nonsignificant reduction in risk of restenosis after coronary angioplasty (level 3 [lacking direct] evidence) - increasing fish consumption may be associated with decreased risk for coronary heart disease and stroke (level 2 [mid-level] evidence)   ***Opinion*:** *Once again, the use of the database that offers a systematic review to the clinical questions becomes more advantageous. In this case, the "Dynamed" database was equally efficient instead of searching the VHL portal with isolated articles.*  *A relaxed atmosphere regarding the food preference of the fish fillet in the daily life of each participant in the workshop, which gave greater importance to the studied subject.* |
| **Table 17: And then, let us use the Evidence Based Health portal? ...** | |
| **Location: Basic Health Unit of Vargem Grande** | |
| **Date and Suggested Activity** | **Results** |
| **12/03/13**  **Discussion of the proposed topics - Vargem Grande** | Attending: All.  We built a flow chart with the participation of the preceptor (see in Thesis) from the results offered in the Workshops already held and we discussed the differentiated importance of studying with the Evidence Based Health portal. Verification of the possibility of much broader studies on the use of database like Dynamed BMJ and Pro quest.  A very important aspect: In relation to the use of Telehealth, the Interns and the preceptor did not show as much interest (they found it interesting), but would be "plastered" (in the students' word) because it works with fixed and vertical subjects (not interactive) in the form of courses.  Scheduled the discussion on the validity of the use of vitamin supplements in the day to day. We opted for the next Workshop in the UNIFESO Library, as the Unit was in great demand of patients and disrupting the discussions. |

| **Table 18: Do you buy multivitamins? On the other hand, would be better to buy fruits, vegetables, vegetables? ...** | |
| --- | --- |
| **Location: Campus of UNIFESO - Library** | |
| **Date and suggested activity** | **Results** |
| **03/22/13**  **Discussion of proposed topics** | Attending: Preceptor and two students.  Internal checked on vitamins and benefits in the BMJ database of the SBE Portal and realized that vitamins can be beneficial in hormonal disorders, skin and mucosal diseases. It was perceived by all that the use of the portal seems to facilitate VERY the perception about the existence of evidence on the chosen themes.  Another inmate had difficulty because the articles presented with the term vitamins were usually associated with their overdosage and then investigated in the VHL the therapeutic use of the vitamins. He cited as an example the use of antineoplastic medications that would cause nephrotoxicity in addition to ovarian cancer testicle and greater susceptibility to develop pulmonary respiratory infections and the fight against the effect of these adversities with the use of Vitamin A, Selenium in addition to amino acids, proving that the use of vitamins , trace elements and amino acids would combat this nephrotoxicity. As she had difficulty finding other articles on the therapeutic use of vitamins, she used google portal and google scholar. He used UERJ (abusive use of multivitamins) as an example of the multivitamin commercialized as Centrium®, noting that "supplementation" is already done by good daily food (fruits, vegetables and vegetables ...) and that only the idea would be valid of vitamin supplementation to those who had, in fact, indications for such, as in the case of intestinal absorption deficit. |

| **Table 19: Application of post-test ...** | |
| --- | --- |
| **Location: Basic Health Unit of Vargem Grande** | |
| Date and suggested activity | **Results** |
| 03/25/13  **Application of the post test with the Likert scale** | Attending: Preceptor and all students.  As the Unit was very crowded and with the application of tests for nursing students in the Unit itself, it was decided to apply the Likert scale test to verify the final results with this 1^st^ group of students, since they will already leave in two weeks for the Elective Internship of duration of 03 months. The final discussion of multivitamins and the use of calcium for treatment of osteoporosis and osteopenia was scheduled. |

| **Table 20: Using the Digital Bookstore and summaries of Cochrane reviews translated into the study of vitamins ...** | |
| --- | --- |
| **Location: Campus of UNIFESO - Library** | |
| **Date and suggested activity** | **Results** |
| **05/04/13**  **Discussion of proposed topics** | Attendings: Doctorate and 01 student. Preceptor made a commitment.  Doctorate: Search the database of the SBE Portal: Proquest®  **Calcium and risk of Acute Myocardial Infarction (AMI):**  The results of a meta-analysis suggested that the use of oral calcium supplements alone (without vitamin D) might be associated with an increased risk of myocardial infarction. However, none of the 15 included studies had cardiovascular outcomes at the primary end-points, and collection of data on cardiovascular events was not standardized. In addition, the results of this analysis may not apply to the use of calcium supplements with vitamin D, which is generally recommended in the treatment and prevention of osteoporosis.  Search the Atheneu Bookstore:  Systematic review offer  **CLINICAL ISSUE**  Does routine use of multivitamins help prevent cancer?  **CONTEXT:** In this meeting, although the students did not attend and for the first time the preceptor can not attend, the great potential of the use of the systematic reviews offered by Cochrane through the library of the Atheneu Library in Health Portal Evidence-Based Library offers several revisions ready for study , which although it was not literally the specific topic previously chosen would be very close to the argument of the same, such as: "Calcium supplementation during pregnancy for prevention of hypertensive disease and associated problems" or "Vitamin A supplementation for post- childbirth "present in Cochrane review summaries, broadly translated as follows: *In the case of Calcium supplementation.*  "Calcium supplementation helps prevent pre-eclampsia, premature birth and decrease the risk of a woman dying or having serious problems related to high blood pressure in pregnancy. Pre-eclampsia is one of the leading causes of death in pregnant women and newborns around the world. Preterm birth (birth before 37 weeks) is often caused by high blood pressure and is one of the leading causes of infant mortality, particularly in low-income countries. A review of 13 studies involving 15,730 women demonstrated that calcium supplementation during pregnancy is a safe and relatively inexpensive way to reduce the risk of preeclampsia in women at increased risk and women in low-calcium diet communities. Women also had a lower chance of dying or having serious problems due to pre-eclampsia. Babies were less likely to be born prematurely. No adverse effects have been found, but further research is needed for the optimal dosage for supplementation. "In the case of vitamin A supplementation: None of the studies was able to demonstrate an effect on the decline in infant mortality, and only a small study showed that child health has improved.  Already also in Cochrane reviews referring to Evidence for the practice in Health - EPAS- the following result was obtained in the study: Multivitamins in the prevention of skin cancer:  There was no difference in the incidence of skin cancer in both groups, and the difference was slightly more pronounced in the prevention of other types of cancer than in the prostate, HR=0.88 (95% CI: 0.79 to 0.98, p=0.02). Multivitamin had no effect on mortality risk. This effect was not observed in men with a family history of cancer and was particularly severe in men who already had a history of cancer at the start of the study.  Opinion: In this way, Cochrane systematic reviews are an integral part of Evidence-Based Health studies and can be a great source of reference for health professionals. |

| **Table 21: A new group of Interns arrives at the Unit ...** | |
| --- | --- |
| **Location: Basic Health Unit of Vargem Grande** | |
| **Date and suggested activity** | **Results** |
| **17/04/13**  **Started a new phase of research with the 2nd group of students of the Internship in Family Health**  **Application of the Pre-test with the Likert scale**  **Use of the doctoral and preceptor login and password to access the SBE Portal** | Attending: Preceptor  Students: 05 Internists  Presentation of the Research Project. Application of the Pre-test with the Likert scale. Talk about Evidence Based Medicine (MBE)  Proposal to discuss clinical topics and prioritize the use of the portal "Evidence-based health" with the use of a doctoral and preceptor login and password  • Themes chosen from consensus with the group  • Discussion of topics from very practical issues such as a TV show call. Elemental questions that include broader questions. It was decided to discuss for the next week according to the option of the group:  • The use of Zinc. What benefits can they bring to health?  • The use of Zinc in childhood, youth and adult life. Are there differences? |

| **Table 22: Zinc? For what? ...** | |
| --- | --- |
| **Location: Campus of UNIFESO - Library** | |
| **Date and suggested activity** | **Results** |
| **24/04/13**  **Discussion of proposed topics** | Attending: All Students and the Preceptor  Discussion on the use of zinc  Doctorate:  Search the database of Best Practice. Dynamed did not yield any results in the search with the term zinc or still associated with vitamins or supplementation and not even with letter Z alone.  From the search in the database of Best Practice came then, "zinc deficiency":  *Zinc deficiency is a lack of sufficient zinc to maintain optimal health, and may have genetic, nutritional, or metabolic etiologies. Zinc is a key micronutrient important in growth and development,* ***immune function****, taste, smell, wound healing, protein synthesis, and maintenance of skin and hair.*   - *Severe zinc deficiency is rare and usually congenital. Milder zinc deficiency is usually acquired and is common in older people.* - *Manifestations of zinc deficiency may be subtle and can affect many organ systems. Delayed wound healing, impaired taste, loss of appetite, hair loss, fertility issues, and increased susceptibility to infection are common manifestations.* - *Plasma or serum zinc levels are useful in the evaluation of patients with suspected zinc deficiency. Milder forms of zinc deficiency may not be detected in plasma or serum tests, but supplementation may still be considered for patients with typical symptoms.* - *In most cases, standard oral zinc supplementation leads to increased zinc levels and amelioration of symptoms.* - *Zinc supplementation is generally safe, although acute toxicity with high doses may lead to adverse changes in immune, iron, copper, and cholesterol status, as well as to potential genital-urinary problems.* - *• Regarding supplementation for children, it is clear that zinc intake is essential and that supplementation for adults is 30 to 40 mg daily. Below is a table with the necessary supplementation in terms of the natural foods of the daily diet:*   [*http://bestpractice.bmj.com/best-practice/monograph/1195/diagnosis/history-and-examination.html*](http://bestpractice.bmj.com/best-practice/monograph/1195/diagnosis/history-and-examination.html)  ***Zinc deficiency***  *Has been known of for 40 years but ignored by global health organizations*  *1^st^ Internist: Supplementation should be with use of 10 mg per 02 to 03 weeks in the age group up to 04 years.*  *2^nd^ Internist: Another reference in which the adult should use an association of Zinc with Iron and for the infant should be supplied from 06 months to 02 years (both Fe and Zn)*  *3^rd^ Internist: In developing countries zinc deficiency appeared in those who did not (or still do not) ingest meats.*  *4^th^ Internist and Preceptor: They studied aspects of metabolism and immunity affected by zinc deficiency.*  *Discussion for the next step of the treatments based on pumpkin seeds for worm diseases and chamomile tea to calm the agitated children.* |

| **Table 23: Can you treat worm diseases (verminose) with pumpkin seed? And chamomile tea calms? ...** | |
| --- | --- |
| **Location: Campus of UNIFESO - Library** | |
| **Date and suggested activity** | **Results** |
| **08/05/13 e 15/05/13**  **Discussion on the use of pumpkin seeds, phytotherapy and some applications of folk medicine, complementary or alternative in the treatment of common complaints in Primary Care** | Initial discussion with an Intern, because two were ill and one missed the tutor absent, because he would be taking a Course this May.  It was exposed by doctoral research on portals on the use of pumpkin seeds, phytotherapy and some applications of folk medicine, complementary or alternative in the treatment of common complaints in Primary Care:  Search for pumpkin seeds without results. Option to look for evidence based answers. These important and interesting answers would already be ready, and although they did not respond directly to the doubts raised, they demonstrated the effectiveness of phytotherapy as a treatment (which is quite interesting since there were doubts about the evidences of this type of treatment). In this way the research in: *"Evidence-based responses" to "Primary Care Problems" in the Evidence-****Based Health portal:***  *These questions pointed out that there is rather evidence regarding phytotherapy treatment for dysmenorrhea, for example: (though not within the reach of the Brazilian public because it is a Chinese tea and achievable from treatments with Chinese medicine)*  *1) How effective is rue tea for the treatment of dysmenorrhea and menorrhagia?*  *It has been found that TOKI-SHAKUYAKU-SAN, a blend of 6 herbs (including angelica and paeonia) - widely used in traditional Chinese medicine - actually has a beneficial effect for the treatment of dysmenorrhea (Grade A) (1).*  *2) There is an indication of the use of chamomile tea, among others in the treatment of lower limb wounds. Is there any evidence as to the effectiveness of this alternative treatment?*  *To date there are no studies to prove its degree of scientific evidence, therefore it is not recommended to use chamomile tea in lesions with their active principles.*  *3) Is it possible to treat insomnia with herbal remedies?*  *No studies were found to indicate elements that recommend or contraindicate the use of medicinal plants for the treatment of insomnia.*  *The research from the VHL has already verified some responses not to the use of pumpkin as a vermifuge, but as a treatment for postmenopausal women:* **Improvement in HDL cholesterol in postmenopausal women supplemented with pumpkin seed oil: pilot study.**  Author(s): Gossell-Williams M; Hyde C; Hunter T; Simms-Stewart D; Fletcher H; McGrowder D; Walters CA  RESULTS: Women receiving pumpkin seed oil showed a significant increase in high-density lipoprotein cholesterol concentrations (0.92 Ã‚Â±0.23 mmol/l vs. 1.07 Ã‚Â±0.27 mmol/l; p=0.029) and decrease in diastolic blood pressure (81.1 Ã‚Â±7.94 mmHg vs. 75.67 Ã‚Â±11.93 mmHg; p < 0.046). There was also a significant improvement in the menopausal symptom scores (18.1 Ã‚Â± 9.0 vs. 13.2 Ã‚Â± 6.7; p < 0.030), with a decrease in severity of hot flushes, less headaches and less joint pains being the main contributors. Women in the group receiving wheat germ oil reported being more depressed and having more unloved feeling.  **CONCLUSION:** This pilot study showed pumpkin seed oil had some benefits for postmenopausal women and provided strong evidence to support further studies.  **A randomized trial of peppermint gel, lanolin ointment, and placebo gel to prevent nipple crack in primiparous breastfeeding women.**  **Fonte: Med Sci Monit; 13(9): CR406-411, 2007 Sep.**  RESULTS: The study groups were comparable in mean age and route of delivery. Nipple crack were less in mothers who received peppermint gel than in those who received lanolin ointment or placebo (chi(2)=16.8, df=6, P=0.01). Relative risk of nipple crack in the lanolin group (RR: 2.41, 95%CI: 1.20-3.01) was higher than in the peppermint group (RR: 1.85, 95%CI: 1.64-3.10). **CONCLUSIONS:** Prophylactic peppermint gel in breastfeeding lactating women is associated with fewer nipple cracks and is more effective than lanolin and placebo. It could be recommended for preventing of nipple crack along with teaching better breastfeeding technique at the initiation of breastfeeding.  *An Internist searched for the term "oil" in Best Practice and ended up checking for the nut oil seed translated into English: peanut oil because he could not find only pumpkin seed oil. It would be used for idiopathic constipation in young adults (20 years). Efficacy not recognized. When searched in the database Best Practice checked Topics under systematic review ready. Found it very interesting*  *He had tried sunflower oil and / or vitamin C and found that there were improvements in venous wounds and ulcers. He verified two more types of oil for specific treatments:*  *Chestnut seed oil that presents improvements for Chronic Venous Insufficiency mainly for pain but not for scarring and.*  *Primrose oil (flower) that would decrease pre menstruation tension (low evidence) although when compared to oophorectomy (removal of the ovaries) the result is lower. He also studied that the association of Primrose oil to fish oil for patients with low risk of preeclampsia would not find satisfactory results in the reduction of this disease.*  *As we only had the presence of an intern, we chose to continue the theme the following week.* |

| **Table 24: Continuing with herbal medicine ...** | |
| --- | --- |
| **Location: Campus of UNIFESO - Library** | |
| **Date and suggested activity** | **Results** |
| **29/05/13**  **Discussion of the proposed theme** | Two Internist (the one from the previous week this time did not attend) presents and the preceptor. One internist verified the use of pumpkin seed and the other the sunflower seed to combat worms disease - once in studying the "pumpkin seed" this term ends up "leading" to the "oil" of this respective seed and by extension also to the oil of the "sunflower seed"; the two classified as vegetable oils.  *The 1^s^t internist verified in the VHL that a study regarding the use of pumpkin seed as vermifuge in children from 02 to 05 years old with use of 05 grams daily of oil, would not have had effectiveness, in fact, effectiveness of 15% as vermifuge which would have been a very low percentage. In relation to the other internal, this verified that the pumpkin seed would lower the levels of triglycerides and of glucose. They found the subject very interesting.*  *The 2^nd^ internist, also from articles in the VHL, found that pumpkin seed could be used in cardiovascular diseases and in venous ulcer healing and protection of gastric mucosa for rats, but that such efficacy was not confirmed for humans. However, the protein efficiency of the sunflower seed (as a food supplement) has been proven.*  *The preceptor was unable to study the proposed theme this time.*  *Theme for the next Workshop:*  *Vertigo: What is it? Etiology and its diagnosis. How do you treat it? Efficacy and indications of cinarizine (anti-dizziness) and Ginkgo biloba (phytotherapeutic used as anti-dizziness)?* |
| **Table 25: Vertigo and anti dizziness ...** | |
| **Location: Campus of UNIFESO - Library** | |
| **Date and suggested activity** | **Results** |
| **05/06/13**  **Discussion of the proposed theme** | Vertigo: What is it? Etiology and its diagnosis. How do you treat it? Efficacy and indications of cinarizine (anti-dizziness) and Ginkgo biloba (phytotherapeutic used as anti-dizziness)?  Doctorate: Search in Dynamed with vertigo and dizzness synthetic and interesting information, but much summarized. Talk with two internists and the preceptor attends later.  In the L Atheneu database there were not many answers (there were no chrocane reviews with vertigo or dizziness)  Biloba G study for BMJ medicines: Excellent study with almost all MBE-based responses, which may make this database widely used for the study of any drug.  VHL Telehealth Research Very good 419 studies being 183 in vertigo.  **Article chosen:**  From dizziness to vertigo: a proposal for the management of the vertiginous patient in Primary Care / From dizziness to vertigo: a proposal for the management of the vertiginous patient in Primary Care  Source: Rev. APS; 11 (1): 62-73, Jan.-Mar. 2008. ilus, tab.  *Vertigo is the illusion of spinning around the environment or vice versa. It is commonly and usually evaluated by the primary care physician (MAP). Some MAPs underestimate it, hence this proposal. A bibliographic survey was carried out in PubMed, LILACS, SciELO, Cochrane, and libraries of the Federal University of Paraná and Pontifícia Universidade Católica do Paraná. It is fundamental knowledge of the vestibular apparatus, divided into peripheral (apparatus and vestibular nerve) and central (nuclei, pathways and cortical vestibular centers). The propaedeutics allows to differentiate vertigo from syncope, imbalance and varied cephalic sensations, besides peripheral (PV) and central (CV) vertigo. PV may be associated with tinnitus, hearing loss, intense vertigo, prostration, sweating and pallor. The nystagmus is exhaustible, horizontal, rotatory or mixed and disappears when fixing the eye. VC is characterized by latency and tolerability of vertigo and is not accompanied by hypoacusis or tinnitus. It can be associated with ataxia, dysarthria, diplopia, sensory, motor or cranial pairs. Nystagmus is inexhaustible, with a different direction in each eye, and shaking in any direction other than horizontal. Complementary exams are unnecessary, except in the cases of CV and Ménière's disease. The treatment is directed to the cause, and the patient should be reassured about the character, most of the time, benign and limited. Symptomatic drugs should be used rationally and for as short a time as possible. We emphasize the need for field research in primary care that would allow us to establish strategies and protocols in the management of vertigo.* |

| **Table 26: PSF tensions. New administration and layoffs in sight ...** | |
| --- | --- |
| **Location: Campus of UNIFESO - Library** | |
| **Date and suggested activity** | **Results** |
| **13/06/13**  **Discussion of the proposed theme and tensions in the administration of the municipality's PSF** | Rediscussion on vertigo. Three Interns present. There was little participation of these and the preceptor can not attend, because he was in an update course covers conducts in Primary Care and Community Therapy to be applied in the BHFU.There is a moment of great tension and possibility of withdrawal of participation of the Pilot Unit, once the Public Bidding for Public Competition of the Management of the PSF of Teresópolis is opened. According to the Group's comment, the firm that currently administers the PSF should dismiss all so that it can be hired by UNIFESO (if it is the winner). This aspect generates discontent and tensions besides the withdrawal of performances of these professionals in the BHFUs in which they are loaded. Faced with this, we schedule the post test with all Interns for next week and we opted for the closing of this Working Group. |

| **Table 27: Application of the post-test. UNIFESO wins the competition. There will be a new contest and ... layoffs on the teams** | |
| --- | --- |
| **Location: Campus of UNIFESO - Library** | |
| **Date and suggested activity** | **Results** |
| **18/06/13**  **Application of the post-test. UNIFESO winning. Tensions in teams with programmed layoffs.**  **Reformulation of all teams**  **Option for completing the Workshops**  **Study of applied questionnaires** | We applied the post-test. Results showed gains in the use of electronic portals. Closing of G2 Group. There was planning with the preceptor to begin activities with the G3 Group of Interns at the BHFU of Vargem Grande for August 2013 or the 3rd week of July (because there are vacations in July). We discussed the possibility of realizing the Project with the participation of the same preceptor. However, this aspect would depend on its approval in the PSF Competition of UNIFESO.  This condition did not occur (there was withdrawal of the application of this preceptor for the Contest).  There was restructuring of all teams, which made it difficult to carry out new workshops.  We decided to close new workshops and, therefore, to apply new questionnaires.  We proceeded to analyze the results presented in the questionnaires already applied. |
